# Oral candidiasis is a significant predictor of subsequent severe infections during immunosuppressive therapy in anti-neutrophil cytoplasmic antibody-associated vasculitis

**DOI:** 10.1186/s12879-019-4300-0

**Published:** 2019-07-26

**Authors:** Makoto Yamaguchi, Takayuki Katsuno, Shiho Iwagaitsu, Hironobu Nobata, Hiroshi Kinashi, Shogo Banno, Yasuhiko Ito

**Affiliations:** Department of Nephrology and Rheumatology, Aichi Medical University, 1-1 Karimata, Yazako, Nagakute, 480-1195 Japan

**Keywords:** Antineutrophil cytoplasmic antibody, Small-vessel vasculitis, ANCA, AAV, Oral candidiasis, Infection

## Abstract

**Background:**

Several studies have identified predictors of severe infections in antineutrophil cytoplasmic antibody-associated vasculitis (AAV). However, the development of oral candidiasis (OC) as a predictor of subsequent severe infections has not been evaluated. The aim of this study was to assess the association between OC and subsequent severe infection requiring hospitalization during immunosuppressive therapy in AAV.

**Methods:**

This single-center retrospective cohort study included 71 consecutive patients with newly diagnosed AAV from Aichi Medical University Hospital, Japan, starting immunosuppressive therapy between March 2013 and December 2018. The relationships between OC and subsequent severe infections were assessed using multivariate Cox proportional hazards models, adjusted for clinically relevant factors.

**Results:**

During the follow-up period (median, 23 months; interquartile range, 11–51 months), 25 severe infectious episodes occurred in 19 patients (26.8%) and OC occurred in 17 patients (23.9%). A log-rank test showed that the OC group was significantly associated with severe infection (*P* <  0.001). Multivariate Cox proportional hazards models identified lower serum albumin (per 1 g/dl adjusted hazard ratio (HR)  = 0.38, 95% confidence interval (CI): 0.15–0.85; *P*  =  0.018), use of methylprednisolone pulse (adjusted HR  =  5.44, 95% CI: 1.54–20.0; *P*  =  0.010), and OC (adjusted HR  = 5.31, 95% CI: 1.86–15.8; *P*  =  0.002) as significant predictors of severe infection. Furthermore, a significant effect modification of the use of methylprednisolone pulse on OC was observed (*P* <  0.001).

**Conclusions:**

OC is one of the predictors of subsequent severe infections. The results suggest the importance of prolonging infection surveillance, especially for patients who developed OC under strong immunosuppressive therapy.

**Electronic supplementary material:**

The online version of this article (10.1186/s12879-019-4300-0) contains supplementary material, which is available to authorized users.

## Background

Antineutrophil cytoplasmic antibody (ANCA)-associated vasculitis (AAV) is a group of systemic vasculitides including microscopic polyangiitis (MPA), granulomatosis with polyangiitis (GPA), and eosinophilic granulomatosis with polyangiitis (EGPA). The diagnosis of AAV is based on the presence of clinical manifestations with characteristic histopathological findings and the presence of myeloperoxidase ANCA (MPO-ANCA) or proteinase 3 ANCA (PR3-ANCA) [1–6]. AAV may affect important organ, which predisposed patients to life-threatening organ failure, such as necrotizing glomerulonephritis and pulmonary involvement [7–9]. The recent advances in immunosuppressive therapy, such as cyclophosphamide (CYC) or rituximab (RTX) in addition to glucocorticoid therapy, have improved the mortality rate of AAV [10–14]. However, the infection rate during treatment has not decreased, and higher mobility and mortality are possible in case of severe infection, especially in elderly patients [1–5, 11–17]. Therefore, it is important to prevent and reduce the risk for developing severe infection.

Although several studies have reported that older age, smoking, worsened kidney function, low level of CD4+ T cells, glucocorticoid, and CYC therapy are significant predictors of severe infection [15–17], other predisposing factors remain unidentified. To the best of our knowledge, no previous studies have focused on the association between oral candidiasis (OC) and subsequent severe infections occurring with AAV therapy and have investigated the incidence of OC in patients undergoing AAV therapy. The occurrence of OC might be a sign of cell-mediated immune decline; thus, we hypothesized that OC might be a predictor of subsequent severe infection in AAV. The aim of this study was to assess the association between OC and subsequent severe infection during immunosuppressive therapy in AAV.

## Methods

### Patients

Our retrospective cohort study included patients aged > 20 years diagnosed with AAV including GPA, MPA, and EGPA on the basis of the European Medicines Agency algorithm [18] with a consensus methodology for the classification of the AAV between March 2013 and December 2018 at Aichi Medical University Hospital. The exclusion criteria were as follows: patients who had been started on immunosuppressive therapy for AAV at another hospital or patients receiving no immunosuppressive therapy. The study protocol was approved by the Ethics Committees of Aichi Medical University (approval number 2018-H350). The requirement for informed consent was waived given the retrospective nature of the study.

### Measurements

The clinical characteristics at the time of starting immunosuppressive treatment were used as baseline, including age, sex, serum creatinine (Scr) level, eGFR [mL/min/1.73 m^2^] = 194 × Scr^_1.094^ × age^_0.287^ × 0.739 [if female] [19])), serum albumin level, C-reactive protein, Birmingham Vasculitis Activity Score (BVAS) 2003 [20], organ involvement, anti-MPO and anti-PR3 ANCA titers, OC, and immunosuppressive treatment; induction immunosuppressive therapy; use of methylprednisolone pulse therapy (0.5 or 1.0 g/d for 3 consecutive days), glucocorticoid monotherapy, intravenous CYC and RTX, maintenance immunosuppressive therapy, glucocorticoid monotherapy, oral CYC, azathioprine, methotrexate, and RTX; point and cumulative prednisolone (PSL) dose; concurrent use of other immunosuppressants at 0, 3, 6, 12, and 24 months after initial immunosuppressive therapy; and adverse events including severe infection during the follow-up period.

All serum samples were tested by direct antigen-specific enzyme-linked immunosorbent assays (ELISAs) for MPO- and PR3-ANCA with serially diluted serum, as previously described [21]. The samples were diluted 1:500 (Nipro Medical Corporation) or 1:101 (Medical and Biological Laboratories Co. Ltd.).

### Outcomes

In this study, the main exposure was the development of OC. OC was defined as clinical signs of oral thrush treated with antifungal drugs, such as itraconazole or fluconazole. The main outcome of interest was the development of severe infection. Severe infection was defined as infection requiring hospitalization. Remission was defined as the absence of clinical signs and symptoms of active vasculitis (BVAS = 0) for more than 2 months. A relapse was defined as clinical signs of vasculitic activity in any organ system following remission [22]. The patients were divided into those who developed OC (OC group) and those who did not (non-OC group). Patients were followed until September 2018 and censored at the time of death (if before primary outcome).

### Statistical analysis

Differences in clinical characteristics between the OC group and the non-OC group were compared by using the Wilcoxon rank-sum test or Fisher’s exact test. To evaluate predictors of severe infection, univariate and multivariate Cox proportional hazards (CPH) models were constructed, including age, sex, serum albumin level, serum creatinine level, methylprednisolone pulse, and OC.

To assess whether an association between methylprednisolone therapy and outcome was different in the OC and non-OC groups, the effect modification between methylprednisolone pulse therapy and the OC group was assessed by the inclusion of interaction terms in the multivariate Cox proportional hazards models.

The proportional hazards assumption for covariates was tested using scaled Schoenfeld residuals. For continuous variables, the Wilcoxon rank-sum test was performed to evaluate the significance of intergroup differences. Categorical variables were expressed as percentages and compared using Fisher’s exact test. The cumulative probability of the development of first severe infection was calculated using the Kaplan-Meier method and log-rank test. The level of statistical significance was set at *P* < 0.05. All statistical analyses were performed using JMP version 14.0.0 (SAS Institute, Cary, NC, USA) and STATA version 13.0 (StataCorp LP, College Station, Texas).

## Results

### Baseline characteristics

From a total of 77 such patients, we excluded 5 (6.5%) patients who had been on immunosuppressive therapy in another hospital and 1 (1.3%) patient with a lack of immunosuppressive therapy. Finally, 71 (92%) consecutive patients with newly diagnosed AAV who were on immunosuppressive therapy were included in this study. The study population consisted of 71 AAV patients, including those with MPA (*n* = 58), GPA (*n* = 1), and EGPA (*n* = 12). During a median observation period of 23 months (interquartile range, 11–51 months), 17 (23.9%) patients developed OC (OC group) after median of 13 days (interquartile range: 11–19 days) of starting immunosuppressive therapy, and 54 (76.1%) had never developed OC (non-OC group). The clinical characteristics of the two groups (OC-group and non-OC group) are summarized in Table 1.

**Table 1:** Clinical characteristics of 71 patients with AAV

	Non-OC (*n* = 54)	OC (*n* = 17)	*P* value
Baseline characteristics			
Age (year)	73 (65–78)	71 (67–76)	0.576
Male (N (%))	30 (55.6)	9 (52.9)	0.850
Serum albumin (mg/dL)	3.0 (2.3–3.7)	2.8 (2.2–3.2)	0.138
Serum creatinine (mg/dL)	0.9 (0.7–1.7)	1.2 (0.5–2.1)	0.925
CRP (mg/dL)	3.3 (0.9–9.0)	4.8 (2.7–9.4)	0.196
IgG (mg/dL)	1682 (1403–2046)	1674 (1170–1992)	0.454
Diagnosis			
MPA	45 (83.3)	13 (76.5)	0.196
GPA	0 (0.0)	1 (5.9)	
EGPA	9 (16.7)	3 (17.6)	
Antibody			
MPO-ANCA	50 (92.6)	17 (100)	0.513
PR3-ANCA	1 (1.9)	0 (0)	
ANCA-negative	3 (5.6)	0 (0)	
BVAS	13 (11–16)	15 (13–16)	0.183
General	52 (96.3)	17 (100)	0.421
Cutaneous	6 (11.1)	5 (29.4)	0.069
Ear, nose, and throat	11 (20.4)	6 (35.3)	0.209
Chest	26 (48.2)	6 (35.3)	0.353
Cardiovascular	1 (1.9)	1 (5.9)	0.381
Abdominal	3 (5.6)	0 (0)	0.321
Renal	28 (51.9)	10 (58.8)	0.615
Nervous system	17 (31.5)	8 (47.1)	0.241
Induction immunosupressive therapy			
Glucocorticoid monotherapy	45 (83.3)	14 (82.4)	0.939
Intravenous cyclophosphamide	5 (9.3)	2 (11.8)	
Rituximab	4 (7.4)	1 (5.9)	
Use of mPSL pulse therapy	18 (33.3)	8 (47.6)	0.306
Maintenance immunosuppressive therapy			
Glucocorticoid monotherapy	34 (63.0)	9 (52.9)	0.863
Oral cyclophosphamide	2 (3.7)	1 (5.9)	
Azathioprine	13 (24.1)	5 (29.4)	
Methotrexate	1 (1.9)	1 (5.9)	
Rituximab	4 (7.4)	1 (5.9)	
Outcomes			
Remission	52 (96.3)	15 (88.2)	0.209
Relapse	19 (38.0)	10 (62.5)	0.086
Hemodialysis	2 (3.7)	2 (11.8)	0.209
Deaths	4 (7.4)	1 (5.9)	0.830
Severe Infection	8 (14.8)	11 (64.7)	< 0.001
Observation period (months)	26 (12–51)	15 (5–47)	0.396

Median (interquartile range), categorical values are expressed as number (proportion)

For continuous variables, the Wilcoxon rank-sum test was performed to assess the significance of inter-group differences

Categorical variables were expressed as percentages and compared by using Fisher’s exact test

*ANCA* anti-neutrophil cytoplasmic antibody, *AAV* ANCA-associated vasculitis, *MPA* microscopic polyangiitis *GPA* granulomatosis with polyangiitis, *EGPA* eosinophilic granulomatosis with polyangiitis, *BVAS* Birmingham Vasculitis Activity Score, *MPO* myeloperoxidase, *PR3* proteinase-3, *OC* oral candidiasis, *mPSL* methylprednisolone

Baseline characteristics were not different significantly between the OC and non-OC groups. Methylprednisolone pulse therapy was administered in 15 (27.8%) and 9 (52.9%) patients in the non-OC and OC groups, respectively (*P* = 0.078).

### Outcomes

#### Remission, relapse, end-stage renal disease, and death

During the observation period, 52 (96.3%) and 15 (88.2%) patients in the non-OC and OC groups achieved remission, respectively (*P* = 0.209). After achieving remission, 19 (38.0%) and 10 (62.5%) patients in the non-OC and OC groups developed relapse, respectively (*P* = 0.086) (Table 1).

During the observation period, 2 (3.7%) patients in the non-OC group and 2 (11.8%) patients in the OC group developed end-stage renal disease requiring permanent dialysis therapy. Furthermore, 4 (7.4%) patients in the non-OC group and 1 (5.9%) patient in the OC group died, and the causes of death were infection (*n* = 3), CVD (*n* = 1), and unknown (*n* = 1).

#### Oral candidiasis and severe infection

During the observation period, a total of 25 severe infection episodes occurred in 19 patients (26.8%), wherein some patients had several infection episodes. The median time from immunosuppressive therapy initiation to the occurrence of first severe infection was 13 months (interquartile range: 7–26 months) in the OC group and 16 months (interquartile range: 1–49 months) in the non-OC group. In the OC group, all severe infections occurred after an OC episode at a median of 2.5 months (interquartile range: 0.3–15 months).

**Fig. 1:**
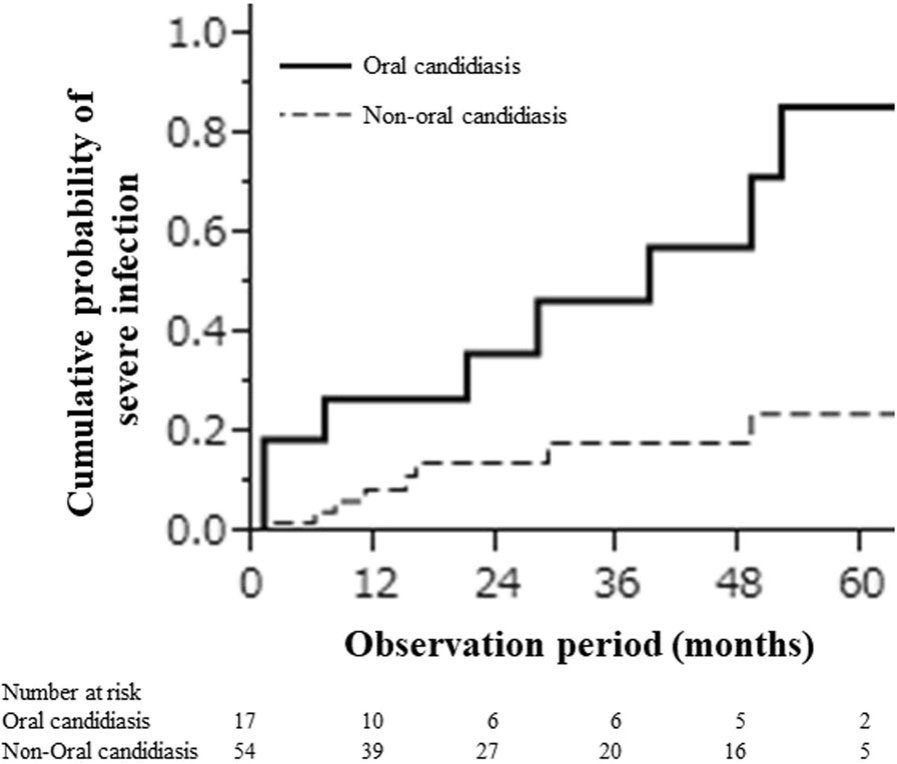
The proportion of patients who developed severe infection was significantly higher [11 (64.7%) vs 8 (14.8%)] in the OC group than in the non-OC group (P < 0.001). The cumulative probabilities of severe infection within 6, 12, and 24 months were 0.18, 0.33, and 0.49 for the OC group and 0.02, 0.08, and 0.05 for the non-OC group, respectively, that is, significant differences in the occurrence of severe infection were found in the OC and non-OC groups (P < 0.001; Fig. 1)

The proportion of patients who developed severe infection was significantly higher [11 (64.7%) vs 8 (14.8%)] in the OC group than in the non-OC group (*P* < 0.001). The cumulative probabilities of severe infection within 6, 12, and 24 months were 0.18, 0.33, and 0.49 for the OC group and 0.02, 0.08, and 0.05 for the non-OC group, respectively, that is, significant differences in the occurrence of severe infection were found in the OC and non-OC groups (*P*  <  0.001; Fig. 1). Severe infections were bacterial pneumonia (*n* =  7), *Pneumocystis jiroveci* pneumonia (*n* =  1), vertebral osteomyelitis (*n* =  1), methicillin-resistant *Staphylococcus aureus* bacteremia (*n* =  1), invasive aspergillosis (*n* =  3), miliary tuberculosis (*n* = 1), fungemia (*n* =  1), and pyelonephritis (*n* =  4).

#### Predictors of severe infections

Predictors of severe infection were evaluated using clinical data including age, sex, serum creatinine level, serum albumin level, use of methylprednisolone pulse, and OC occurrence on the basis of previous reports [15–17, 22–24]. Univariate CPH analyses identified several statistically significant predictors of increased infection risk, namely, lower serum albumin level, higher serum creatinine level, use of methylprednisolone pulse, and OC (Table 2). A multivariate CPH model adjusted for age, sex, serum creatinine, serum albumin, use of methylprednisolone pulse, and OC identified lower serum albumin (per 1 g/dl adjusted hazard ratio (HR)  = 0.38, 95% confidence interval (CI): 0.15–0.85; *P*  =  0.018), use of methylprednisolone pulse (adjusted HR  =  5.44, 95% CI: 1.54–20.0; *P*  =  0.010), and OC (adjusted HR  = 5.31, 95% CI: 1.86–15.8; *P*  =  0.002) as significant predictors. Furthermore, the effect modification of the use of methylprednisolone pulse on OC was observed (*P* < 0.001).

**Table 2:** Predictors of severe infection

	Univariate model		Multivariate model	
	HR (95% CI)	*P* value	HR (95% CI)	*P* value
Age (per 10 years)	1.03 (0.99–1.09)	0.134	1.05 (0.99–1.12)	0.071
Male (vs. female)	1.02 (0.41–2.53)	0.959	1.61 (0.55–4.76)	0.387
Serum albumin (per 1.0 g/dL)	0.41 (0.21–0.78)	0.007	0.45 (0.19–0.99)	0.045
Serum creatinine (per 1.0 mg/dL)	1.31 (1.02–1.63)	0.038	1.16 (0.85–1.59)	0.371
Methylprednisolone pulse therapy	2.71 (1.08–6.78)	0.033	3.59 (1.07–12.0)	0.038
Oral candidiasis	5.34 (2.13–13.4)	< 0.001	4.55 (1.73–12.0)	0.002

*HR* hazard ratio, *CI* confidence interval

Data are the HR, 95% CI, and *P* value from Cox proportional hazard regression analyses

Adjusted for baseline characteristics including age, sex, serum albumin level, serum creatinine level, methylprednisolone pulse therapy, and oral candidiasis

During the observation period before the occurrence of severe infection (or censoring without outcome), no significant difference was observed between the OC and non-OC groups in terms of the point and cumulative PSL dose, and using immunosuppressants at 0, 3, 6, 12, and 24 months after initiating immunosuppressive therapy (Table 3). These data suggest that differences in the intensity of immunosuppressive therapy between OC and non-OC groups did not explain the differences in the occurrence of severe infection.

**Table 3:** Immunosuppressive treatment during the observation period

	Non-OC	OC	*P* value
Baseline	(*n* = 54)	(*n* = 17)	
Prednisolone (mg/kg/day)	0.91 (0.69–1.15)	1.01 (0.86–1.23)	0.109
3rd month	(*n* = 49)	(*n* = 16)	
Prednisolone (mg/kg/day)	0.34 (0.27–0.40)	0.31 (0.24–0.40)	0.537
Cumulative dose of prednisolone (g)	1.6 (1.4–1.8)	1.8 (1.6–1.9)	0.312
Use of immunosuppressants [n (%)]	7 (14.3)	4 (25.0)	0.309
6th month	(*n* = 47)	(*n* = 12)	
Prednisolone (mg/kg/day)	0.24 (0.2–0.30)	0.21 (0.21–0.30)	0.605
Cumulative dose of prednisolone (g)	2.6 (2.2–3.0)	2.5 (2.2–3.0)	0.936
Use of immunosuppressants [n (%)]	9 (19.1)	3 (17.6)	0.811
1st year	(*n* = 39)	(*n* = 10)	
Prednisolone (mg/kg/day)	0.18 (0.13–0.20)	0.19 (0.16–0.28)	0.189
Cumulative dose of prednisolone (g)	3.5 (2.9–4.0)	3.2 (2.6–3.8)	0.442
Use of immunosuppressants [n (%)]	10 (25.6)	4 (40.0)	0.517
2nd year	(*n* = 28)	(*n* = 6)	
Prednisolone (mg/kg/day)	0.15 (0.12–0.18)	0.17 (0.13–0.25)	0.320
Cumulative dose of prednisolone (g)	4.8 (3.7–5.9)	3.9 (2.7–6.2)	0.473
Use of immunosuppressants [n (%)]	9 (32.1)	4 (66.7)	0.474

Median (interquartile range), categorical values are expressed as number (proportion)

For continuous variables, the Wilcoxon rank-sum test was performed to assess the significance of inter-group differences

Categorical variables were expressed as percentages and compared by using Fisher’s exact test

## Discussion

This study revealed that OC was significantly associated with subsequent severe infection, after adjusting for important preventable risk factors, suggesting that OC might be a significant predictor of subsequent severe infection. Furthermore, significant effect modifications between OC and methylprednisolone pulse showed that those who developed OC under strong immunosuppressive treatment were more vulnerable to severe infections. This finding suggested that physicians should be careful in treating OC patients, especially those under aggressive immunosuppressive treatment, to allow for earlier detection and better outcome. This study had some advantages. First, to the best of our knowledge, no previous studies have focused on OC as a predictor of severe infection. Furthermore, details of immunosuppressive treatment during the follow-up period were assessed.

Previous studies revealed that AAV patients who developed severe infection during immunosuppressive treatment are associated with a high mortality [1–5, 11–17, 22–27], and the main injured organ due to infection was the lungs [23]. Several risk factors for severe infections have been identified, including older age at diagnosis, severe kidney involvement at diagnosis, low level of CD4^+^ T cell, and immunosuppressive treatment using high-dose glucocorticoid and CY [11–17, 22–27]. Although our result was compatible with those of previous studies, which showed that patients who use high-dose glucocorticoids such as methylprednisolone pulse therapy were at a higher risk for severe infection, no previous studies have focused on the association between OC and subsequent severe infection in AAV.

OC is a common opportunistic infection of the oral cavity caused by an overgrowth of *Candida* species, and the most common is *Candida albicans* [28]. The reported risk factors for OC were broad-spectrum antibiotics, immunosuppressive drugs, smoking, diabetes, Cushing’s syndrome, immunosuppressive conditions such as human immunodeficiency virus infection, malignancies such as leukemia, and nutritional deficiencies [28]. In this study, we considered that in AAV patients, OC might be an important sign of decreased cellular immune function, predisposing the patient further to subsequent severe infections. This study also showed that glucocorticoid dose and use of immunosuppressive treatment during the observation period were clinically comparative between the OC and non-OC groups, suggesting that differences in the intensity of immunosuppressive therapy between OC and non-OC did not explain the differences in the occurrence of severe infections. Interestingly, this study also showed an interaction between OC and methylprednisolone pulse therapy, suggesting that patients who developed OC under strong immunosuppressive therapy might have an increased risk of severe infection.

Regarding OC treatment, although all patients were prescribed oral antifungal drugs such as itraconazole or fluconazole for systemic effects, we consider that OC treatment itself does not directly influence the outcome of severe infection.

As immunosuppressive therapy, glucocorticoid monotherapy was frequently used for the treatment of AAV in the present study based on previous Japanese studies [29, 30]. Although a recent study showed that glucocorticoid monotherapy is considered less effective than combination therapy with an immunosuppressive agent, such as CYC or RTX, in other countries, it is unknown whether elderly MPA patients who might be at a high risk of infection should use these aggressive immunosuppressive therapies. In our cohort, glucocorticoid monotherapy was frequently used for elderly patients considering the risk of infection; therefore, we could not evaluate the relationship between IVCY or RTX therapy and severe infection because the number of patients administered with IVCY or RTX was insufficient to evaluate it. This should be considered when interpreting our results.

This study also showed that lower serum albumin level was a significant risk factor for severe infection in AAV patients, as previously reported [24]. The present study suggests that patients with AAV presenting hypoalbuminemia should be carefully managed for severe infections.

Our study has some limitations. First, given the retrospective nature of this study, we should consider that unmeasured factors associated with the treatment may not be included in the model. Second, this study has a single-center small cohort design and the observation period was short; therefore, our results should be validated in other multicenter large cohorts with longer follow-ups. Third, in Japan, most AAV patients were elderly MPA patients; thus, the subjects in this study may not be representative of all AAV patients. Fourth, several studies recommend CYC or RTX therapy for AAV patients as induction therapy [10–14]. However, few patients in our study were treated with RTX or CYC. Therefore, we could not assess the influence of RTX or CYC on the outcome; a larger cohort using the treatment should be included to re-evaluate our results. Thus, we advise caution when interpreting and generalizing our results. Despite these methodological issues, to the best of our knowledge, this study is the first to describe the relationship between OC and subsequent severe infection in AAV patients.

## Conclusions

OC is one of the predictors of subsequent severe infections. The results suggest the importance of prolonging infection surveillance, especially for patients who developed OC under strong immunosuppressive therapy.

## Additional file

Additional file 1: The anonymous data set of 71 patients with AAV. (XLSX 22 kb)

## Data Availability

The corresponding author had full access to all of the data in the study and had final responsibility for the decision to submit the manuscript for publication. Materials described in the manuscript, including all relevant data, will be freely available to any scientist wishing to use them for non-commercial purposes, without breaching participant confidentiality through requesting to this email: yasuito@aichi-med-u.ac.jp

## Abbreviations

AAV: Antineutrophil cytoplasmic antibody-associated vasculitis
ANCA: Antineutrophil cytoplasmic antibody
CYC: Cyclophosphamide
EGPA: Eosinophilic granulomatosis with polyangiitis
GPA: Granulomatosis with polyangiitis
MPA: Microscopic polyangiitis
OC: Oral candidiasis
RTX: Rituximab

## References

[1] Reinhold-Keller E, Beuge N, Latza U, de Groot K, Rudert H, Nölle B (2000). An interdisciplinary approach to the care of patients with Wegener’s granulomatosis long-term outcome in 155 patients. Arthritis Rheum.

[2] Lazarus B, John GT, O’Callaghan C, Ranganathan D (2016). Recent advances in anti-neutrophil cytoplasmic antibody-associated vasculitis. Indian J Nephrol.

[3] Li ZY, Ma TT, Chen M, Zhao MH (2015). The prevalence and management of anti-neutrophil cytoplasmic antibody-associated vasculitis in China. Kidney Dis (Basel).

[4] Alberici F, Martorana D, Vaglio A (2015). Genetic aspects of anti-neutrophil cytoplasmic antibody-associated vasculitis. Nephrol Dial Transplant.

[5] Charlier C, Henegar C, Launay O, Pagnoux C, Berezné A, Bienvenu B (2009). Risk factors for major infections in Wegener granulomatosis: analysis of 113 patients. Ann Rheum Dis.

[6] Gavraud M, Guillevin L, Le Toumelin P, Cohen P, Lhote F, Casassus P (2001). Long-term followup of polyarteritis nodosa, microscopic polyangiitis, and Churg-Strauss syndrome. Analysis of four prospective trials including 278 patients. Arthritis Rheum.

[7] Youssef J, Novosad SA, Winthrop KL (2016). Infection risk and safety of corticosteroid use. Rheum Dis Clin N Am.

[8] Kallenberg CG (2014). The diagnosis and classification of microscopic polyangiitis. J Autoimmun.

[9] Sinico RA, Meroni P (2013). The kaleidoscopic manifestations of systemic vasculitis. Autoimmun Rev.

[10] Harper L, Savage CO (2005). ANCA-associated renal vasculitis at the end of the twentieth century-a disease of older patients. Rheumatology.

[11] Mukhtyar C, Guillevin L, Cid MC, Dasgupta B, de Groot K, Gross W (2009). EULAR recommendations for the management of primary small and medium vessel vasculitis. Ann Rheum Dis.

[12] Flossmann O, Berden A, de Groot K, Hagen C, Harper L, Heijl C (2011). Long-term patient survival in ANCA-associated vasculitis. Ann Rheum Dis.

[13] Jones RB, Tervaert JW, Hauser T, Luqmani R, Morgan MD, Peh CA (2010). Rituximab versus cyclophosphamide in ANCA-associated renal vasculitis. N Engl J Med.

[14] Stone JH, Merkel PA, Spiera R, Seo P, Langford CA, Hoffman GS (2010). Rituximab versus cyclophosphamide for ANCA-associated renal vasculitis. N Engl J Med.

[15] Kronbichler A, Jayne DR, Mayer G (2015). Frequency, risk factors and prophylaxis of infection in ANCA-associated vasculitis. Eur J Clin Investig.

[16] McGregor JC, Negrete-Lopez R, Poulton CJ, Kidd JM, Katsanos SL, Goetz L (2015). Adverse events and infectious burden, microbes and temporal outline from immunosuppressive therapy in antineutrophil cytoplasmic antibody-associated vasculitis with native renal function. Nephrol Dial Transplant.

[17] Yang L, Xie H, Liu Z, Chen Y, Wang J, Zhang H (2018). Risk factors for infectious complications of ANCA-associated vasculitis: a cohort study. BMC Nephrol.

[18] Watts R, Lane S, Hanslik T, Hauser T, Hellmich B, Koldingsnes W (2007). Development and validation of a consensus methodology for the classification of the ANCA-associated vasculitides and polyarteritis nodosa for epidemiological studies. Ann Rheum Dis.

[19] Matsuo S, Imai E, Horio M, Yasuda Y, Tomita K, Nitta K (2009). Collaborators developing the Japanese equation for estimated GFR. Revised equations for estimated GFR from serum creatinine in Japan. Am J Kidney Dis.

[20] Luqmani RA, Bacon PA, Moots RJ, Janssen BA, Pall A, Emery P (1994). Birmingham Vasculitis activity score (BVAS) in systemic necrotizing vasculitis. QJM..

[21] Ito-Ihara T, Muso E, Kobayashi S, Uno K, Tamura N, Yamanishi Y (2008). A comparative study of the diagnostic accuracy of ELISA systems for the detection of anti-neutrophil cytoplasm antibodies available in Japan and Europe. Clin Exp Rheumatol.

[22] Hogan SL, Falk RJ, Chin H, Cai J, Jennette CE, Jennette JC (2005). Predictors of relapse and treatment resistance in antineutrophil cytoplasmic antibody–associated small-vessel vasculitis. Ann Intern Med.

[23] Kitagawa K, Furuichi K, Sagara A, Shinozaki Y, Kitajima S, Toyama T (2016). Risk factors associated with relapse or infectious complications in Japanese patients with microscopic polyangiitis. Clin Exp Nephrol.

[24] Xu PC, Tong ZY, Chen T, Gao S, Hu SY, Yang XW (2018). Hypoalbuminaemia in antineutrophil cytoplasmic antibody-associated vasculitis: incidence and significance. Clin Exp Rheumatol.

[25] Mohammad AJ, Segelmark M, Smith R, Englund M, Nilsson JÅ, Westman K (2017). Severe infection in antineutrophil cytoplasmic antibody-associated vasculitis. J Rheumatol.

[26] Little MA, Nightingale P, Verburgh CA, Hauser T, De Groot K, Savage C (2010). Early mortality in systemic vasculitis: relative contribution of adverse events and active vasculitis. Ann Rheum Dis.

[27] Dalrymple LS, Go AS (2008). Epidemiology of acute infections among patients with chronic kidney disease. Clin J Am Soc Nephrol.

[28] Singh A, Verma R, Murari A, Agrawal A (2014). Oral candidiasis: an overview. J Oral Maxillofac Pathol.

[29] Yamagata K, Usui J, Saito C, Yamaguchi N, Hirayama K, Mase K (2012). ANCA-associated systemic vasculitis in Japan: clinical features and prognostic changes. Clin Exp Nephrol.

[30] Harigai M, Nagasaka K, Amano K, Bando M, Dobashi H, Kawakami T (2019). 2017 clinical practice guidelines of the Japan research Committee of the Ministry of health, labour, and welfare for intractable Vasculitis for the management of ANCA-associated vasculitis. Mod Rheumatol.

